# Anti-Tumor Active Isopropylated Fused Azaisocytosine-Containing Congeners Are Safe for Developing *Danio rerio* as Well as Red Blood Cells and Activate Apoptotic Caspases in Human Breast Carcinoma Cells

**DOI:** 10.3390/molecules27041211

**Published:** 2022-02-11

**Authors:** Małgorzata Sztanke, Jolanta Rzymowska, Krzysztof Sztanke

**Affiliations:** 1 Chair and Department of Medical Chemistry, Medical University of Lublin, 4A Chodźki Street, 20-093 Lublin, Poland; 2 Department of Biology and Genetics, Medical University of Lublin, 4A Chodźki Street, 20-093 Lublin, Poland; jolanta.rzymowska@umlub.pl; 3 Laboratory of Bioorganic Synthesis and Analysis, Chair and Department of Medical Chemistry, Medical University of Lublin, 4A Chodźki Street, 20-093 Lublin, Poland; krzysztof.sztanke@umlub.pl

**Keywords:** isopropylated fused azaisocytosine-containing congeners, in vivo zebrafish embryo acute toxicity study, phenotypic parameters, developmental abnormalities, structure–toxicity relationships, hemolysis assay, apoptotic caspase activation

## Abstract

New isopropylated fused azaisocytosine-containing congeners (**I–VI**) have previously been reported as promising anticancer drug candidates, so further research on these molecules in the preclinical development phase is fully justified and necessary. For this reason, in the present paper, we assess the toxicity/safety profiles of all the compounds using *Danio rerio* and red blood cell models, and examine the effect of the most selective congeners on the activation of apoptotic caspases in cancer and normal cells. In order to evaluate the effect of each molecule on the development of zebrafish embryos/larvae and to select the safest compounds for further study, various phenotypic parameters (i.e., mortality, hatchability, heart rate, heart oedema, yolk sac utilization, swim bladder development and body shape) were observed, and the half maximal lethal concentration, the maximal non-lethal concentration and no observed adverse effect concentration for each compound were established. The effect of all the isopropylated molecules was compared to that of an anticancer agent pemetrexed. The lipophilicity-dependent structure–toxicity correlations were also determined. To establish the possible interaction of the compounds with red blood cells, an ex vivo hemolysis test was performed. It was shown that almost all of the investigated isopropylated congeners have no adverse phenotypic effect on zebrafish development during five-day exposure at concentrations up to 50 μM (**I**–**III**) or up to 20 μM (**IV**–**V**), and that they are less toxic for embryos/larvae than pemetrexed, demonstrating their safety. At the same time, all the molecules did not adversely affect the red blood cells, which confirms their very good hemocompatibility. Moreover, they proved to be activators of apoptotic caspases, as they increased caspase-3, -7 and -9 levels in human breast carcinoma cells. The conducted research allows us to select—from among the anticancer active drug candidates—compounds that are safe for developing zebrafish and red blood cells, suitable for further in vivo pharmacological tests.

## 1. Introduction

**Figure 1:**
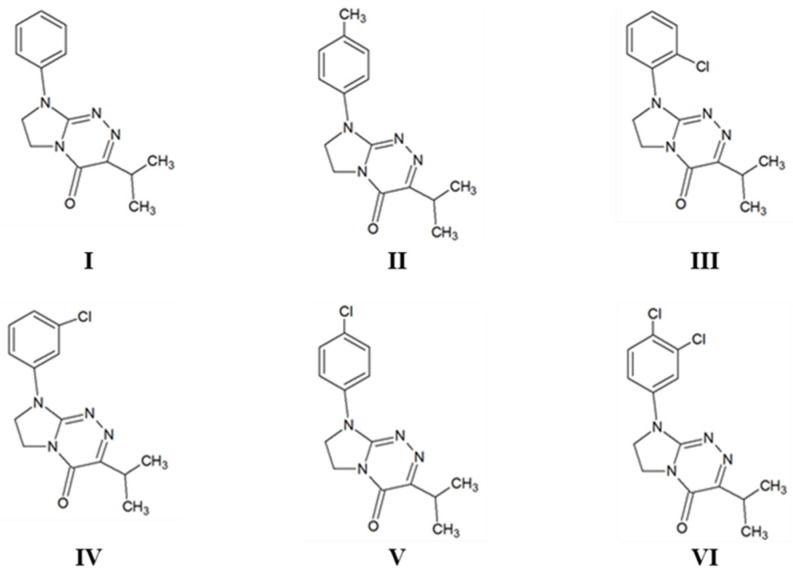
Structures of the studied compounds (**I**–**VI**). These molecules are ordered in relation to the presence of different substituent attached at the *N*8 of the privileged scaffold as follows: the phenyl derivative, i.e., a parent structure (**I**), *para*-methylphenyl derivative (**II**), *ortho*-chlorophenyl derivative (**III**), *meta*-chlorophenyl derivative (**IV**), *para*-chlorophenyl derivative (**V**) and 3,4-dichlorophenyl derivative (**VI**). All the investigated congeners possess the common privileged scaffold of 7,8-dihydroimidazo[2,1-*c*][1,2,4]triazin-4(6*H*)-one and the isopropyl substitution at *C*3.

We reported in our earlier paper [1] a novel class of isopropylated fused azaisocytosine-containing congeners (**I**–**VI**, Figure 1), which was synthesized in our laboratory as a result of the fruitful isosteric replacement. In this way, promising drug candidates were obtained which—being isosteres of endogenic nucleobases—can act as irreversible inactivators of specific enzymes (antimetabolites). Due to the remarkable in vitro antiproliferative effect against human tumors of the breast, cervix and lung—even stronger than that of an anticancer agent pemetrexed—and a lower toxicity to normal cells, the title compounds are of particular pharmacological importance. In addition, most of these isopropylated congeners show the protective effect on oxidatively-stressed erythrocytes, better or comparable to that of ascorbic acid and Trolox, and they are characterized by lipophilicity indices ensuring the optimal absorption and effective permeability in body compartments [1,2]. It is hoped that the most selective molecules of this class will find application in cancer chemotherapy in the future.

Taking into account the significant pharmacological activities, selectivity as well as favorable pharmacokinetic and physico-chemical properties of novel isopropylated fused azaisocytosine-containing congeners—recently patented [3] and published [1,2]—further studies on these compounds were fully justified. As the development of highly safe drugs is desirable, each newly synthesized potential drug candidate should be carefully assessed for its toxicity/safety in an animal model already at an early stage of its preclinical development, before entering the clinical trials [4,5,6]. One of the appropriate research models is the recently very popular organism—the zebrafish (*Danio rerio*). This small fish is an attractive alternative to research on other vertebrates, such as mice and rats. As a model organism, the zebrafish has a number of advantages, such as the small size, high fecundity, rapid development ex utero, transparency of the body in the early stages of development, high genetic homology to humans, and easy and inexpensive breeding [4,7]. According to European Union legislation [8], studies on zebrafish up to the fifth day after fertilization do not require permission for animal testing. *Danio rerio* is a valuable tool—with a proven accuracy in predicting toxicity in humans—for the screening of various compounds. Thanks to the research using this vertebrate model, it is possible to evaluate the toxicity/safety of the newly synthesized promising molecules—potential drug candidates [4,7]. Zebrafish, at the early stage of development, are very useful in these studies because developing embryos are more susceptible to chemical compounds than adults, which makes assays using embryos more sensitive. In turn, the ex utero development and body transparency allow for the detailed observation of the compound-induced effects without killing the maternal organism. The administration of the test compound is also easy as embryos absorb the molecules from the water [4,5,9,10]. All these features make *Danio rerio* a suitable model to study many aspects of toxicity in the preclinical phase of drug development.

Due to the fact that the isopropylated fused azaisocytosine-containing congeners can be considered valuable anticancer drug candidates, further extensive research on this novel class of molecules was required. To date, none of these compounds have been screened for toxicity/safety using an animal model, so the zebrafish was an excellent modern tool for their testing. Therefore, the main aim of this study is to evaluate the effect of the promising isopropylated molecules on the development of zebrafish embryos/larvae and to select the safest compounds for further research. To achieve this, a wide range of morphological traits (including some endpoints for the assessment of toxicity in mammals [11]) that are commonly affected by exposure to chemicals during development were assessed. Furthermore, the half maximal lethal concentration (LC_50_), the maximal non-lethal concentration (MNLC) and no observed adverse effect concentration (NOAEC) values for each test molecule were determined. The effect of the title congeners on the embryonic and larval development of zebrafish was compared (in the same experimental model) with the effect of an anticancer agent pemetrexed. In addition, the correlations between the structure of the compounds (resulting from their different substitutions at the *N*8) and their toxicity to zebrafish were established. Moreover, the relationship between the lipophilicity and toxicity of each compound was confirmed. Studies assessing—for the first time—the safety profile of the title compounds for zebrafish are very important because the toxicological characteristics of these molecules in this vertebrate model are helpful in selecting the safest isopropylated fused azaisocytosine-containing congeners for further in vivo testing in mammals.

Toxicological screening using the most abundant body cells, i.e., red blood cells, is another important test to assess the toxicity/safety profile of potential drug candidates in the early stage of preclinical development. Mammalian erythrocytes are widely used for studying the cytotoxicity due to the availability and simplicity of cell isolation, as well as the similarity of the red blood cell membrane to other cell membranes. This erythrocyte model enables the direct assessment of the toxicity of intravenous preparations, as well as a general evaluation of the toxicity of the newly synthesized compounds, which—by interacting with the cell membrane—can alter its permeability and thus promote hemolysis [12]. The assessment of the hemocompatibility of compounds in the preclinical development is of great importance for their further studies in an in vivo model. Therefore, another goal of our study is to determine the possible interaction of the newly synthesized isopropylated congeners with red blood cells.

Our previous in vitro study [1] showed that the title isopropylated molecules were more effective at inhibiting the proliferation of some human tumor cells than a clinically useful pemetrexed. One of the possible mechanisms by which anti-tumor agents kill cancer cells is apoptosis. Although several different signaling pathways can lead to this programmed cell death, the activation of caspases is a key pathway. Caspases—aspartate-specific cysteine proteases—are activated via the macromolecular signaling complexes, and this activation ultimately results in the programmed execution of cell death, and the nature of this cell death is determined by the specific caspases involved. Due to the role of caspases in apoptosis, they are divided into the initiator and executioner caspases. Initiator caspases (caspase-2, -8, -9 and -10) act as proteolytic signal amplifiers to activate effector caspases (caspase-3, -6 and -7), which proteolytically cleave a number of structural and regulatory proteins, thus facilitating apoptosis. Many anticancer therapies are based on the induction of apoptosis through the indirect involvement of caspases. The search for new synthetic compounds that can specifically activate individual caspases or suppress the natural inhibitors of caspases present in the cell is of great importance for the development of new anticancer therapies [13,14]. Therefore, the next goal of this study is to investigate the effect of isopropylated fused azaisocytosine-containing congeners on the activation of apoptotic caspases in cancer and normal cells.

## 2. Results and Discussion

### 2.1. The Effect of the Tested Isopropylated Fused Azaisocytosine-Containing Congeners (***I***–***VI***) on Developing Zebrafish

The zebrafish shares many cellular and physiological features with higher vertebrates, making it an excellent animal model for testing the acute and chronic toxicity as well as for studying the developmental toxicity of newly synthesized drug candidates [4,5,7,9]. Therefore, in this study, we used 1–5-day-old zebrafish embryos/larvae as animal model organisms and evaluated the toxicity and safety of new promising anticancer agent candidates, i.e., the isopropylated fused azaisocytosine-containing congeners. To assess the adverse effects of test compounds on zebrafish development, we studied observable phenotypic parameters—such as the mortality, ability to hatch, heart rate, heart oedema, yolk sac utilization, swim bladder development and body shape—in embryos/larvae that were exposed to the compounds/pemetrexed at various concentrations. These parameters were monitored and documented daily between 24 and 120 hpf in each group in comparison to the control embryos/larvae, which were not treated with any of the compounds. Moreover, the half maximal lethal concentration (LC_50_), the maximal non-lethal concentration (MNLC) and no observed adverse effect concentration (NOAEC) values for each test molecule and pemetrexed were determined.

The toxicity screening of the compounds showed a dose- and structure-dependent effect on zebrafish embryos/larvae that were exposed to each compound or pemetrexed for five days.

**Figure 2:**
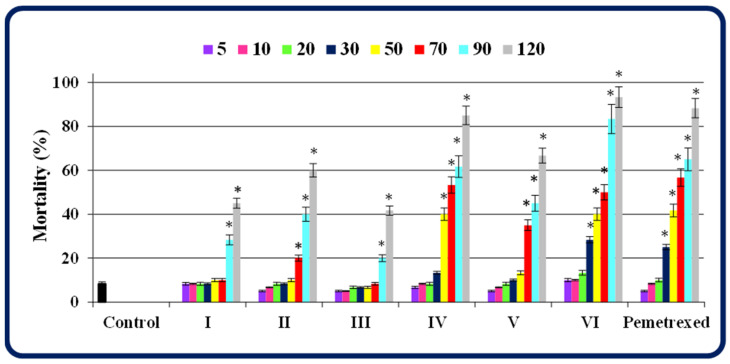
The mortality of zebrafish at the end of the 5-day exposure to test compounds **I**–**VI** and pemetrexed at concentrations of 5, 10, 20, 30, 50, 70, 90 and 120 µM. In groups exposed to the compounds/pemetrexed at a 150 µM concentration, the mortality was 100%. The mortality was calculated as follows: (a number of individuals that did not survive/a number of individuals exposed to a single concentration of the compound/pemetrexed in each experiment) × 100%. Data represent the mean ± SD of three independent experiments with similar experimental conditions. *—statistically significantly different from the control group (*p* < 0.05, Student’s *t*-test).

The isopropylated congeners at concentrations up to 70 μM (**III** and **I**), 50 μM (**II** and **V**), 30 μM (**IV**) or 20 μM (**VI**) did not cause significant mortality in the developing zebrafish—compared to the control—at the end of the 5-day exposure period to the compounds. At the same time, the anticancer drug pemetrexed shows no lethal effects only at concentrations up to 20 μM (Figure 2). Among this class of molecules, the compounds **III**, **I** and **II** caused the lowest mortality of zebrafish, and their maximal non-lethal concentrations were found to be 76.7, 63.3 and 50 μM, respectively. The molecules **V** and **IV** (with the MNLC = 43.3 and 30 μM, respectively) were more toxic than the previous ones. It is worth noting that the MNLC values of all these compounds were higher than that of pemetrexed (the MNLC = 20 μM), indicating their safety for zebrafish. In turn, the most toxic of this class of congeners proved to be the molecule **VI**, showing the maximal non-lethal concentration slightly lower than that of this anticancer agent (Table 1).

**Figure 3:**
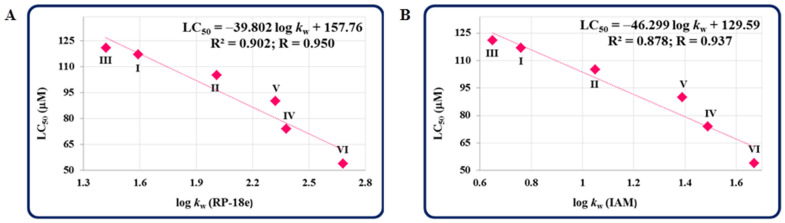
Correlations between the LC_50_ values of the compounds (**I**–**VI**) and their standardized lipophilicity indices (logs *k*_w_) determined on the RP-18e (**A**) and IAM (**B**) columns.

Noteworthy is that the half maximal lethal concentration (LC_50_) values, based on the mortality at the end of a five-day exposure of developing zebrafish to the compounds, were found to be higher for almost all the isopropylated congeners (except **VI**) than for pemetrexed, demonstrating that these molecules are safer for zebrafish than this anticancer agent (Table 1). Additionally, by examining the influence of the lipophilicity of all the compounds on their toxicity, strong correlations between the log *k*_w_ values [1] (determined on two various columns) and the LC_50_ values of the compounds were confirmed, which indicates that the toxicity of novel isopropylated congeners towards developing zebrafish enhances with an increase in their lipophilicity (Figure 3). These observations are consistent with those reported in previous studies on zebrafish embryos [15,16,17].

**Figure 4:**
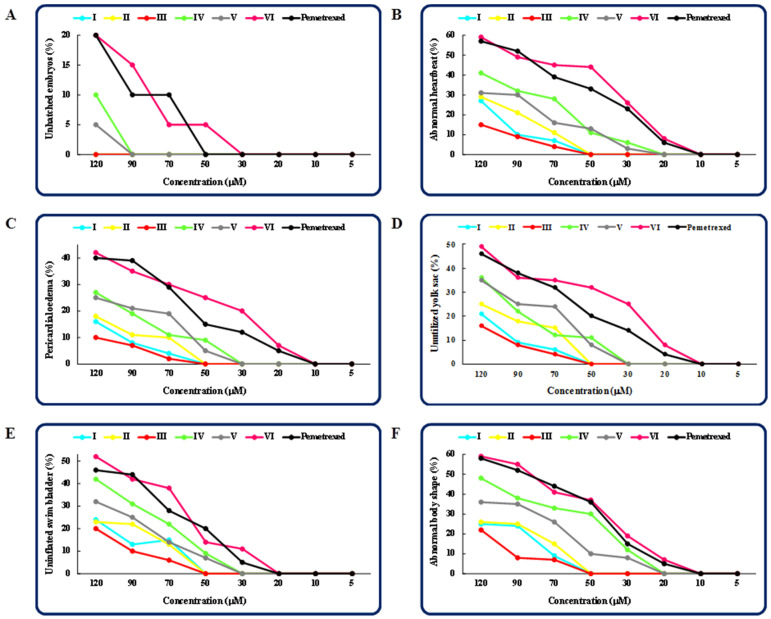
Dose-dependent effects of the compounds **I**–**VI** and pemetrexed on zebrafish after 5-day exposure. The percentage of unhatched embryos in the compound/pemetrexed-treated groups (**A**). The percentage of larvae with an abnormal heartbeat in the compound/pemetrexed-treated groups (**B**). The percentage of larvae with a pericardial oedema in the compound/pemetrexed-treated groups (**C**). The percentage of larvae with an unutilized yolk sac in the compound/pemetrexed-treated groups (**D**). The percentage of larvae with an uninflated swim bladder in the compound/pemetrexed-treated groups (**E**). The percentage of larvae with an abnormal body shape in the compound/pemetrexed-treated groups (**F**).

**Figure 5:**
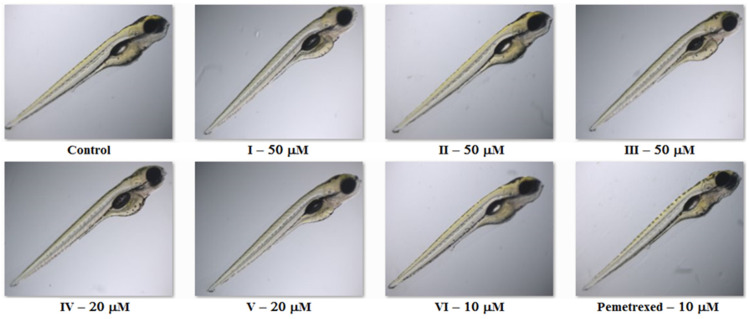
The representative 5-day-old larvae from the control group as well as from groups exposed to the highest concentration of each compound or pemetrexed, which is considered safe, at the end of the observation period.

**Figure 6:**
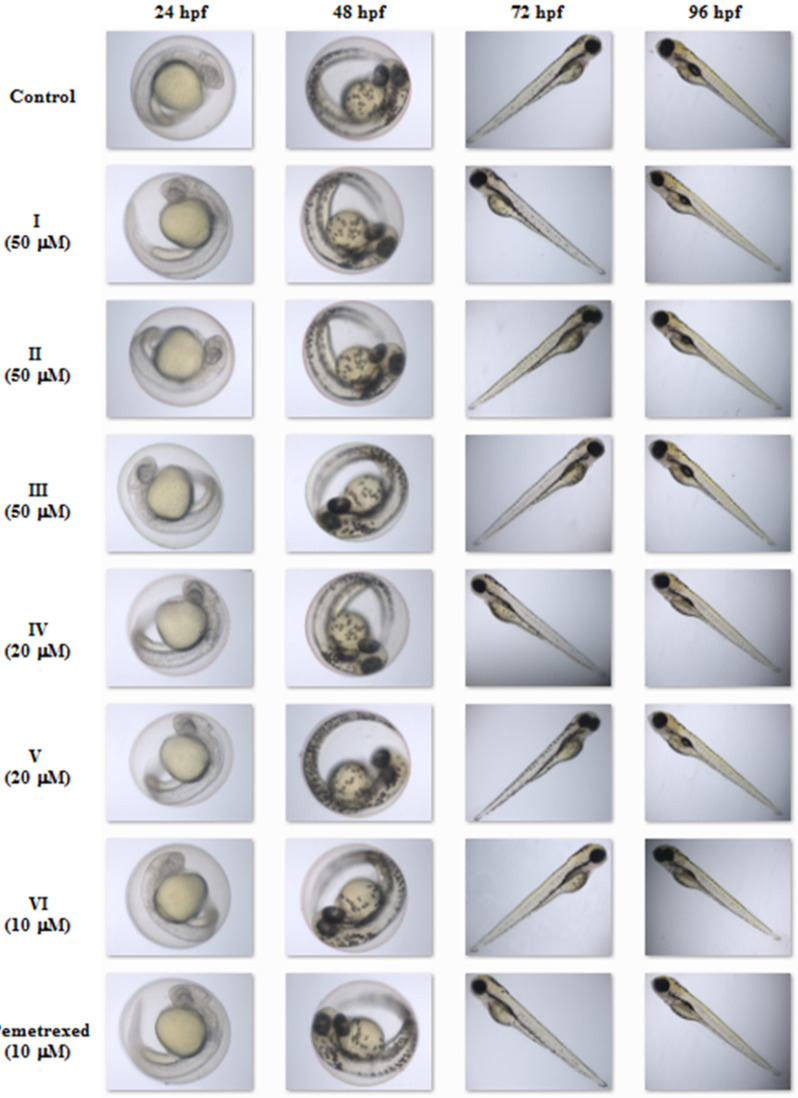
Zebrafish in the control and compound/pemetrexed treated groups at 24, 48, 72 and 96 hpf. The images show representative normally developing control embryos/larvae as well as those treated with the highest concentration of the compound/pemetrexed that did not induce phenotypic abnormalities.

To assess the adverse effects of the investigated molecules on developing zebrafish, we analyzed additional observable phenotypic parameters, namely, hatchability, heart rate, pericardial oedema, utilization of the yolk sac, development of the swim bladder and body shape in embryos/larvae exposed to the compounds/pemetrexed. Figure 4 presents the graphs of the dose-dependent effects (the percentage of unhatched embryos (**A**), the percentage of larvae with an abnormal heartbeat (**B**); the percentage of larvae with a pericardial oedema (**C**); the percentage of larvae with an unutilized yolk sac (**D**); the percentage of larvae with an uninflated swim bladder (**E**); and the percentage of larvae with an abnormal body shape (**F**)) of each compound tested and pemetrexed on zebrafish after 5-day exposure. The highest concentration of the compound/pemetrexed that induced no phenotypic abnormalities in zebrafish larvae—defined as no observed adverse effect concentration (NOAEC) (Table 1)—was considered as safe. Among all the tested isopropylated congeners, three molecules, i.e., **III**, **I** and **II,** proved to be the least toxic, as they did not cause any malformations in developing zebrafish at concentrations up to 50 μM. These compounds had no adverse effect on the ability to hatch, heartbeat rate, heart and swim bladder development, yolk sac utilization or body shape throughout the embryonic development period up to 5 dpf, and the compound-treated larvae developed normally. The compounds **V** and **IV** at or below 30 μM concentrations showed no significant effect on the heart, yolk sac and swim bladder, while at a concentration of 20 μM or lower, they did not cause an abnormal body shape. In turn, the most toxic proved to be the compound **VI**, which at a concentration of 30 μM exhibited an adverse effect on the development of swim bladder, and at a concentration of 20 μM induced oedema of the pericardium and yolk sac, decreased heart rate and caused an abnormal body shape. It is worth noting that although the exposure of zebrafish embryos/larvae to higher concentrations of the compounds **I**–**V** resulted in various developmental abnormalities, these defects were observed less frequently than in embryos/larvae exposed to the clinically useful anticancer drug pemetrexed. These results clearly indicate that the molecules **I**–**V** can be considered safe for zebrafish. The images in Figure 5 show the representative 5-day-old larvae exposed to the highest safe concentration of the compound/pemetrexed that did not induce any observable phenotypic abnormalities. Properly developing embryos/larvae from the groups treated with the compound/pemetrexed at the highest safe concentration and from the control group at 24, 48, 72 and 96 hpf are presented in Figure 6. On the other hand, the most serious phenotypic abnormalities in zebrafish embryos/larvae caused by the highest concentrations of the compound **VI** and pemetrexed are shown in Figure S1 in the Supplementary Material.

The hatching rate is one of the important indicators for assessing the developmental toxicity of pharmacologically important compounds in fish. Under normal conditions, zebrafish embryos hatch between 48 and 72 h. An embryo is considered to have hatched if it has completely left the chorion. Many compounds and drug candidates can affect the hatchability. Various mechanisms, such as a malfunction of the chorionase enzyme, and/or an inability of the embryo to break through the chorion, and/or a growth retardation and inhibition of embryo development, may be responsible for the delay or failure of hatching [18,19,20]. In our study, the majority of zebrafish embryos that were treated with the isopropylated congeners were hatched during the experiment, usually between 48 and 72 hpf. The compounds **I**–**III** at concentrations up to 120 μM, **IV**–**V** up to 90 μM, and **VI** up to 30 μM, while pemetrexed up to 50 μM, had no significant effect on the hatchability. In turn, in each of the remaining groups, only a few percentage of the surviving embryos did not hatch during the observation period (Figure 4A).

The heart is the first organ that forms and functions during organogenesis in zebrafish. It starts beating at about 22 to 26 hpf. The heart rate—based on the number of beats per minute—reflects the heart function, making it an important sub-lethal endpoint in fish embryo toxicity studies. Changes in the heart rate can be a common reaction to exposure to various compounds. Some of them accelerate the heart rate, while others slow it down. An increased heart rate can overload the heart muscle and cause functional damage to the zebrafish heart. In the case of heart malformations, some compounds may induce pericardial oedema in zebrafish embryos. This may be due to increased cell death in the developing heart of treated embryos, and/or an inappropriate activation of intracellular calcium, resulting in reduced myocardial size and increased blood pressure, and/or disturbances in the osmoregulatory system that cause pericardial hyperhydration [18,19,21,22,23,24,25]. Abnormalities in the heart can also be caused by damage to the yolk sac, which serves as a nutritional reservoir for the embryo. The impairment of the yolk sac can block nutrient delivery during the embryonic development. Due to energy deficiency, the heart function may be impaired, which is manifested by a slower heartbeat and the occurrence of various malformations [21]. The swelling of the yolk sac, which is a sign of decreased nutrient absorption by the embryo, is a common abnormality observed in zebrafish toxicity tests. This can be affected by the hyperhydration, osmoregulation and accumulation of toxins in the yolk. Yolk retention is associated with reduced fish mobility and/or yolk malabsorption. On the other hand, a small yolk sac indicates increased movement and/or rapid consumption of the yolk [19]. In the present study, zebrafish larvae did not show a marked heart rate response to exposure to the molecules **I**–**III** at concentrations up to 50 µM, **IV**–**V** up to 20 µM, and **VI** and pemetrexed up to 10 µM (Figure 4B). In contrast, the larvae exposed to higher concentrations of the compounds/pemetrexed had a reduced heart rate compared to the control. Additionally, in larvae treated with the congeners **I**–**III** at concentrations up to 50 µM, **IV**–**V** up to 30 µM, and **VI** and pemetrexed up to 10 µM, no pericardial as well as yolk sac oedemas were observed (Figure 4C,D). It is interesting that in the compound/pemetrexed-treated groups where pericardial oedema had occurred, it was usually accompanied by yolk sac oedema. This indicates that the pericardial swelling may be caused by lesion of the yolk sac. The simultaneous occurrence of these two deformities was frequently found in other studies [21].

The gas-filled swim bladder is another key organ in fish that is often assessed in toxicity tests of the newly synthesized compounds. It allows the fish to float in the water, which minimizes the energy required to maintain a vertical body position. In addition, it protects other visceral organs in the abdomen from damage by external hydraulic pressure and supplies oxygen when the fish is in an anoxic condition. Inflation of the swim bladder occurs early in the larval period 1–3 days after hatching, and is controlled by autonomic reflexes acting on vascular, muscular and secretory effectors [24,26]. In our study, we did not observe any abnormalities of the swim bladder in zebrafish exposed to the compounds **I**–**III** at concentrations up to 50 μM, **IV**–**V** up to 30 μM, and **VI** and pemetrexed up to 20 μM (Figure 4E). In these groups, the swim bladder was gas-filled at the end of the five-day exposure to the compounds/pemetrexed.

Body shape is also a significant parameter in fish for assessing the potential toxicity of drug candidates. Spinal curvature and tail abnormalities are commonly observed developmental toxicity parameters in zebrafish. Spine deformities can be caused by depletion/deregulation of calcium and phosphorus ions or a decrease in myosin levels, which control the body axis formation and spine formation in properly developing zebrafish embryos. These disorders significantly affect the zebrafish motility and make it impossible to swim properly [18,20,23]. In this study, larvae exposed to the compounds **I**–**III** at concentrations up to 50 µM, **IV**–**V** up to 20 µM, and **VI** and pemetrexed up to 10 µM had a normal body shape. A curvature of the spine and/or a tail defect were observed only in groups treated with higher concentrations of the compounds/pemetrexed (Figure 4F).

Analyzing the structure–toxicity relationships, it turned out that among this class of molecules, the safest for developing zebrafish are compounds containing as a part of their structure the 2-chlorophenyl (**III**), phenyl (**I**—the parent structure) and 4-methylphenyl (**II**) substituents at the *N*8. The same monosubstituted (**III**, **II**) and unsubstituted (**I**) derivatives were disclosed as the least lipophilic molecules [1] in a whole set. This is as expected as in general the toxicity of organic compounds decreases with their decreasing lipophilicity [27]. Although two derivatives bearing the 4-chlorophenyl (**V**) and 3-chlorophenyl (**IV**) at the *N*8 were slightly more toxic than the previous ones, they were less toxic than pemetrexed. On the other hand, the compound containing two chlorine atoms at positions 3 and 4 of the phenyl moiety (**VI**) proved to be the most toxic of all the tested congeners. This suggests that monosubstituted (*ortho*-chorophenyl, *para*-methylphenyl, *para*-chlorophenyl and *meta*-chlorophenyl) as well as unsubstituted phenyl moieties are preferred for normal zebrafish development. In contrast, the presence of two chlorine atoms on the phenyl moiety intensified the mortality and developmental abnormalities, and therefore the 3,4-dichlorophenyl substitution is not favorable due to the increased toxicity. This can be explained by the known fact that the toxicity of many organic compounds enhances with their increasing lipophilicity caused by an increase in the number of chlorine atoms in an aromatic phenyl moiety [27].

### 2.2. The Hemolytic Activities of the Investigated Isopropylated Fused Azaisocytosine-Containing Congeners (***I***–***VI***)

Another ex vivo study allowing us to assess the toxicity/safety profile of potential drug candidates at an early stage of their preclinical development is the hemolysis test. Synthetic compounds can directly interact with blood cells, altering the permeability of erythrocyte membranes and thus inducing hemolysis [12]. Due to the promising anti-tumor activity of all the isopropylated fused azaisocytosine-containing congeners (**I**–**VI**), it was worth assessing their hemocompatibility. To verify the toxicity/safety potential for red blood cells, all the compounds were assessed for their hemolytic properties. It is clearly shown that all the studied molecules at a concentration of 200 μM, when incubated with red blood cells, do not induce hemolysis, and therefore they are completely non-toxic to erythrocytes (Table 2). In addition, in our previous study [1], most of the isopropylated congeners were found to be effective in inhibiting the oxidative hemolysis of red blood cells exposed to reactive oxygen species, and their protective effect against oxidative damage to erythrocytes was better or comparable to antioxidant standards, such as ascorbic acid or Trolox. Therefore, it should be emphasized that these compounds (**I**–**VI**)—identified as promising anticancer drug candidates—are safe for red blood cells even at a concentration exceeding their IC_50_ values in the in vitro cytotoxicity test, which is important for their further in vivo testing, as they would not have an adverse effect on the erythrocytes.

### 2.3. The Ability of Compounds ***III***, ***V*** and ***VI*** to Activate Caspase-3, -7 and -9 in Normal Cells and Tumor Cells of the Breast 

**Figure 7:**
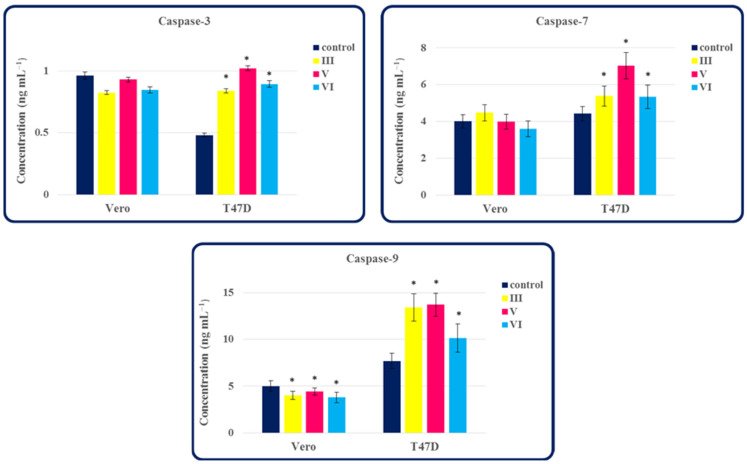
The effect of compounds **III**, **V** and **VI** on the caspase-3, -7 and -9 activation in normal and tumor cells. Normal cell line: Vero (ECACC 88020401)—African Green Monkey kidney cells. Cancer cell line: T47D (ECACC 85102201)—human breast carcinoma cells. Data represent the mean ± SD of three independent experiments. *—statistically significantly different from the control (*p* < 0.05, Student’s *t*-test).

In our previous study [1], the isopropylated fused azaisocytosine-containing congeners proved to be the promising anticancer drug candidates, because they inhibited the in vitro proliferation of various cancerous cells. One possible mechanism for killing cancer cells is through apoptosis, which is initiated and executed by a family of cysteine proteases called caspases [14]. Therefore, it was purposeful to evaluate the effect of chosen compounds on apoptotic caspases in normal and cancer cells. For this purpose, we investigated the ability of anticancer active and selective congeners **III**, **V** and **VI** to activate the caspase-3, -7 and -9 in human breast carcinoma T47D cells and normal Vero cells. Among these caspases, the effector caspase-3 and -7 are key proteins in the execution phase of apoptosis, while an initiator caspase-9 plays a crucial role in the initiation phase of apoptosis [13]. As shown in Figure 7, all the selected compounds (**III**, **V** and **VI**) induce the activation of caspase-3, -7 and -9 in human breast carcinoma cells, as compared to the controls. Treatment of T47D cells with the molecules **III**, **V** and **VI** increased caspase-3 (1.74- to 2.12-fold), caspase-7 (1.21- to 1.59-fold) and caspase-9 (1.32- to 1.78-fold) levels, compared to the untreated controls. In turn, in normal Vero cells, these congeners showed no effect on the activation of the effector caspase-3 and -7, but inhibited the level of an initiator caspase-9. Based on the results above, the isopropylated molecules tested can be considered as activators of caspases, which may be involved in the apoptotic pathway. Breast cancer is the most common malignant tumor among women in the world, therefore scientists are still intensively searching for effective drugs for breast cancer, for example, those that would act through the caspase-dependent apoptosis pathway [14]. Taking into account the obtained results, the investigated anticancer active compounds, as activators of apoptotic caspases, may be promising agents in the field of anticancer drug discovery.

### 2.4. Predicting Molecular Targets for the Isopropylated Fused Azaisocytosine-Containing Congeners (***I***–***VI***)

When searching the Therapeutic Target Database (TTD) (http://db.idrblab.net/ttd/; accessed on 31 January 2022), it was found that none of the isopropylated fused azaisocytosine-containing congeners (**I**–**VI**) has a Tanimoto coefficient above 0.7, meaning that these molecules are not considered structurally similar to any approved or investigational drug. Therefore, most likely, the mechanism of activity of our compounds is not strictly related to the mechanism of action of any known agent.

In order to assess a drug score and the possible molecular targets for the test molecules (**I**–**VI**), the Molinspiration Cheminformatics software (available online at www.molinspiration.com; accessed on 31 January 2022) was employed. All the in silico calculations were also performed for the standard drug used in our research. 

A drug score index is very useful for qualifying the investigated compound as a potential drug candidate. This descriptor is calculated on the basis of the physico-chemical parameters (cLogP, logS, molecular weight), drug-likeness and toxicity risks of the molecule. In general, the higher the drug score, the higher the probability that the compound will be a drug in the future [28]. All the investigated isopropylated congeners (**I**–**VI**) revealed favorable physico-chemical and pharmacokinetic properties and they were devoid of undesired side effects, such as mutagenicity, tumorigenicity, irritating and reproductive effects, as previously reported [2]. It was found that a drug score for compound **VI** was almost the same as that of pemetrexed. In turn, a drug score for molecules **I**–**V** was even higher (in the range of 0.73–0.84) than that of a standard drug (Table 3). This seems to be in agreement with our experimental data obtained in the zebrafish model.

The isopropylated congeners (**I**–**VI**) were evaluated as potential G-protein-coupled receptor (GPCR) ligands, enzyme inhibitors, kinase inhibitors, nuclear receptor ligands and protease inhibitors. Virtual screening has shown that these small molecules may act at different targets, and they have the highest probability to be active as ligands modulating G-protein-coupled receptors (GPCRs) and enzyme inhibitors, according to their bioactivity scores (Table 3). The enzyme inhibition appears to be possible since the studied compounds were designed as fused azaisocytosine-containing congeners as well as nitrogen bridgehead congeners of the purines [1]. Such molecules may be capable of replacing biogenic nucleobases and participating in metabolic pathways [27]. Additionally, taking into account the previous experimental findings concerning structurally related compounds [29,30], one can suppose that the title molecules may reveal antagonistic activity towards adenosine receptors that belong to GPCRs.

## 3. Materials and Methods

### 3.1. The Investigated Compounds (***I***–***VI***)

For our current research needs, all the isopropylated fused azaisocytosine-containing congeners (**I**–**VI**, Figure 1) were resynthesized as substances of high purity following their synthetic methodology reported earlier. Their molecular structures were established on the basis of spectroscopic data and retention times consistent with those presented in the previous paper. The design and synthesis of this novel class of potential innovative antimetabolites, as well as their full structural characterization and pharmacological relevance, have been recently reported [1,3].

### 3.2. In Vivo Studies

#### 3.2.1. Preparation of Solutions of the Tested Compounds

The isopropylated fused azaisocytosine-containing congeners (**I**–**VI**) were assessed for their toxicity and safety to developing zebrafish embryos/larvae. Before beginning the experiment, series of solutions of all the tested compounds were prepared. For this purpose, the investigated molecules were dissolved in dimethyl sulfoxide (DMSO; POCH SA, Gliwice, Poland) to prepare stock solutions, which were then diluted with an embryonic medium (E3 medium: a purified water containing 5 mM NaCl, 0.33 mM MgCl_2_, 0.33 mM CaCl_2_, 0.17 mM KCl and adjusted to pH 7.2) to obtain the appropriate compound concentrations in the range of 5–150 μM (selected on the basis of preliminary screening tests). Pemetrexed (Sigma-Aldrich, Saint Louis, MO, USA)—an anticancer agent—at the same concentrations as the tested compounds was used as a positive control, whereas the embryonic medium—as a negative control. Since DMSO had no embryotoxic effect on zebrafish at all the concentrations used in the preliminary studies, a DMSO control group was not required. All solutions were always freshly prepared just before starting the experiment.

#### 3.2.2. Zebrafish Embryo Acute Toxicity Studies

Acute toxicity studies were based on the OECD (Organization for Economic Cooperation and Development) guidelines for the testing of chemicals [31], and were performed according to the modified procedures described earlier [15,32]. 

All the experiments with the use of zebrafish were carried out in accordance with the EU Directive 2010/63 on the protection of animals used for scientific purposes [8], as well as the applicable Polish legislation. Under these regulations, the self-feeding larval forms of zebrafish up to 120 hpf are not defined as protected and their use for scientific purposes is not subject to regulations on animal studies.

Toxicity studies were carried out at the Experimental Medicine Centre of the Medical University of Lublin, Poland, on zebrafish embryos that were obtained by mating adults of the wild-type AB strain of zebrafish using standard methods [33]. One hour after fertilization, the eggs were collected and placed in Petri dishes (Costar, Corning Inc., Glendale, AZ, USA) containing a fresh embryonic medium. Next, the embryos were rinsed several times in an embryonic medium, and only the viable, normally dividing and spherical embryos were selected and transferred into 12-well sterile cell culture plates (Costar, Corning Inc., Glendale, AZ, USA). In each well, ten embryos were placed in approximately 4 mL of an embryonic medium with or without the compound/pemetrexed. The embryos were exposed to test solutions up to 120 hpf. All the solutions were changed regularly every 24 h and non-viable embryos, dead eggs or chorion remains were removed at the same time. In each group, twenty embryos were exposed to different concentrations (ranging from 5 to 150 μM) of the compounds/pemetrexed. An equal number of embryos untreated with any compound/pemetrexed served as the control. All the covered plates were kept in an incubator under static conditions at 28.5 ± 0.5 °C throughout the experiment. 

To assess the acute toxicity of the investigated compounds (**I**–**VI**) and pemetrexed on zebrafish embryos/larvae, the following observable phenotypic parameters, such as the mortality, ability to hatch, heartbeat, heart oedema, yolk sac utilization, swim bladder development and body shape, were monitored and documented daily between 24 and 120 hpf. Malformations and abnormalities in the compound/pemetrexed-treated groups were compared with the control group. For these observations, the SteREO Discovery.V8 optical microscope with a camera (Zeiss, Göttingen, Germany) was used. The acute toxicity of each compound/pemetrexed was assessed on the basis of a positive outcome of any of the observable phenotypic parameters as well as the established MNLC (the maximal non-lethal concentration), LC_50_ (the half maximal lethal concentration) and NOAEC (no observed adverse effect concentration) values. The MNLC represents the maximum concentration of the compound/pemetrexed that does not result in a statistically different mortality of zebrafish embryos/larvae compared to the control group. The LC_50_, representing the lowest concentration of the compound/pemetrexed at which 50% of the embryos/larvae are dead, was calculated (using the probit method [34]) by fitting the sigmoid curve to mortality data at the end of a five-day exposure of developing zebrafish to the compounds/pemetrexed at different concentrations. The NOAEC is the highest concentration of the compound/pemetrexed at which no adverse effect is observed. Data were obtained from three independent experiments with similar experimental conditions.

### 3.3. Ex Vivo Studies

#### 3.3.1. Red Blood Cells

The blood was collected into heparinized tubes (Li-Heparin: 16 IU mL^−1^) from two male Wistar rats (8–9 weeks old, 200–250 g) kept at the Experimental Medicine Centre, Medical University of Lublin, Poland. After centrifugation (1000 rpm, 10 min, 4 °C) and the removal of the plasma, the erythrocytes were washed three times with phosphate-buffered saline (PBS, pH 7.4, Biomed, Lublin, Poland). A 4% suspension of erythrocytes, obtained by suspending the cells in PBS, was used for the determination of hemolysis.

#### 3.3.2. Hemolysis Assay

The hemolytic activity of the tested compounds (**I**–**VI**) was assessed in the ex vivo model of rat erythrocytes according to the procedure described earlier [35]. Briefly, each test compound at a concentration of 200 μM after mixing with the erythrocyte suspension was incubated in a shaker incubator at 37 °C with mild shaking for 60 min. Then, the samples were centrifuged at 3000 rpm for 10 min, and the absorbance of each supernatant was measured at λ_max_ = 540 nm using a Hitachi U2800 spectrophotometer (Hitachi, Tokyo, Japan). Erythrocytes in PBS solution and in 10% Triton X-100 solution (Sigma-Aldrich, St. Louis, MO, USA) were employed as negative (absence of hemolysis) and positive (100% hemolysis) controls, respectively.

### 3.4. In Vitro Studies

#### Measurement of Caspase-3, -7 and -9 Levels in Normal and Tumor Cells

The caspase-3, -7 and -9 levels were determined—according to the procedure described earlier [15]—using commercially available ELISA kits (CASP3, CASP7, CASP9, SunRed Biotechnology Company, Shanghai, China), which are based on the double-antibody sandwich enzyme-linked immunosorbent assay. Briefly, after preparing the reagents, standards and cell culture supernatants according to the manufacturer’s instructions, both untreated (controls) and the compound-treated (150 μM) lysates of normal (i.e., Vero) or tumor (i.e., T47D) cells were added to the microplate wells, pre-coated with the Human CASP3 or 7 or 9 monoclonal antibodies. Then, biotinylated detection antibodies specific for each caspase and a streptavidin–horseradish peroxidase conjugate was added. After incubation (37 °C, 60 min), the plates were washed five times with a wash buffer to remove the uncombined enzyme, and after the addition of the A and B chromogen solutions, these were further incubated in the dark (37 °C, 10 min). Finally, a stop solution was added to terminate the enzyme-substrate reaction. Optical densities were measured within 10 min on an ELISA reader (BIO-TEK Instruments Inc., Winooski, Vermont, USA) at λ = 405 nm, and the caspase-3, -7 and -9 concentrations were calculated using the standard curve for each caspase.

### 3.5. Statistical Analysis

All the experimental assays were performed in triplicate. The obtained results were averaged and then expressed as the mean ± SD. Statistical analyses of the data were performed using the statistical software Statistica 9.1.PL (StatSoft, Cracow, Poland). Only these values were considered statistically significant, for which *p* was less than 0.05 at a 95% confidence level.

## 4. Conclusions

The results of the in vivo studies—based on comparative studies with pemetrexed—clearly indicate that almost all (with the exception of one) isopropylated fused azaisocytosine-containing congeners can be regarded as safe for developing zebrafish, making them promising drug candidates in the preclinical phase of drug development, and therefore suitable for further pharmacological tests. The five-day exposure of zebrafish embryos/larvae to the tested compounds at concentrations up to 50 μM (molecules **I**–**III**) or up to 20 μM (molecules **IV**–**V**) did not cause a significant effect on any of the parameters assessed, such as the mortality, ability to hatch, heart rate, pericardial oedema, yolk sac utilization, swim bladder development and body shape. It is noteworthy that the clinically useful anticancer drug pemetrexed showed no negative effect on the observable phenotypic parameters in zebrafish, only when administered at a concentration of 10 μM or less. In addition, the maximal non-lethal concentration, the half maximal lethal concentration and no observed adverse effect concentration values for **I**–**V**, established at the end of the five-day exposure of developing zebrafish to these compounds, were found to be higher than those of pemetrexed. Of all the molecules tested, the most preferred structures—with respect to reduced toxicity—were the least lipophilic monosubstituted (especially those bearing the 2-chlorophenyl and 4-methylphenyl) and unsubstituted (i.e., the parent structure) derivatives, which proved to be the safest for the normal development of zebrafish. Furthermore, our ex vivo studies confirmed the very good hemocompatibility of the tested isopropylated congeners, indicating their appropriateness as potential drug candidates. All the molecules did not adversely affect the red blood cells and therefore can be considered as safe for erythrocytes, which is especially important for their further in vivo testing. Moreover, the isopropylated compounds in human breast carcinoma cells proved to be activators of apoptotic caspases that play a key role in both the initiation (caspase-9) and execution (caspase-3 and -7) phases of programmed cell death. Considering the role of caspases in apoptosis, the in vitro anticancer active molecules may prove to be effective in the treatment of human cancers, especially breast cancer.

Studies of the title isopropylated fused azaisocytosine-containing congeners, carried out in both animal and cellular models, allowed us to assess the toxicity/safety profile of these compounds and to determine their effect on apoptotic caspases. This characterization is important to expand and clarify the knowledge of this new class of molecules in the field of preclinical toxicology and pharmacology. From a practical point of view, the conducted research allowed for the selection of compounds—from among all the isopropylated congeners being promising anticancer drug candidates—safe for both *Danio rerio* and red blood cells, which will be submitted for further in vivo pharmacological tests.

## Figures and Tables

**Table 1:** The maximal non-lethal concentration (MNLC), the half maximal lethal concentration (LC_50_) and no observed adverse effect concentration (NOAEC) values of the compounds **I**–**VI** and pemetrexed for zebrafish embryos/larvae.

Compound	MNTC (µM) ^1^	LC_50_ (95% CL ^2^, µM)	NOAEC (µM)
**I**	63.3 ± 11.5	117 (107–129)	50
**II**	50.0 ± 0.0	105 (96–118)	50
**III**	76.7 ± 11.5	121 (115–130)	50
**IV**	30.0 ± 0.0	74 (65–86)	20
**V**	43.3 ± 11.5	90 (79–100)	20
**VI**	16.7 ± 5.8	54 (44–70)	10
Pemetrexed	20.0 ± 0.0	68 (59–78)	10

^1^ The mean ± SD, ^2^ CL—confidence limit.

**Table 2:** Hemolytic activity of the investigated compounds (**I**–**VI**) at a 200 μM concentration.

Compound/Control		Hemolytic Activity (in %)
Compound	**I**	0
	**II**	0
	**III**	0
	**IV**	0
	**V**	0
	**VI**	0
A positive control	10% Triton X-100 solution	100
A negative control	Phosphate Buffered Saline	0

**Table 3:** Prediction of the drug score and bioactivity score for various molecular targets.

Compound/Standard Drug	Drug Score	Bioactivity Score					
		GPCR Ligand	Enzyme Inhibitor	Kinase Inhibitor	Ion Channel Modulator	Nuclear Receptor Ligand	Protease Inhibitor
**I**	0.84	−0.41	−0.37	−0.61	−0.77	−0.95	−1.00
**II**	0.79	−0.40	−0.41	−0.59	−0.82	−0.89	−0.98
**III**	0.73	−0.28	−0.41	−0.40	−0.74	−0.93	−0.98
**IV**	0.73	−0.36	−0.41	−0.55	−0.74	−0.89	−1.00
**V**	0.73	−0.35	−0.39	−0.56	−0.74	−0.88	−0.96
**VI**	0.60	−0.30	−0.39	−0.48	−0.70	−0.80	−0.91
**PMX**	0.58	0.27	0.39	0.45	−0.02	−0.51	0.05

PMX—pemetrexed disodium; GPCR—G-protein-couplet receptor.

## Data Availability

The data presented in this study are available on request from the authors.

## Supplementary Materials

The following are available online, Figure S1: The representative 1-, 2-, 3-, 4- and 5-day-old zebrafish from the control group (**A**–**E**) as well as from groups exposed to the highest concentrations of the compound **VI** (**F**–**J**) or pemetrexed (**K**–**O**).

## Sample Availability

Samples of the compounds **I**–**VI** are available from the authors.
