# Supplementary material for: Anti-Tumor Active Isopropylated Fused Azaisocytosine-Containing Congeners Are Safe for Developing Danio rerio as Well as Red Blood Cells and Activate Apoptotic Caspases in Human Breast Carcinoma Cells
Source: Molecules. 2022 Feb 11;27(4):1211. doi: 10.3390/molecules27041211 (PMC8876100; doi:10.3390/molecules27041211)
Supplement: Supplementary file 1 [file molecules-27-01211-s001.zip › molecules-1581933-supplementary.pdf]

molecules

MDPI

Supplementary Material

Antitumour active isopropylated fused azaisocytosine-containing congeners are safe for developing *Danio rerio* as well as red blood cells and activate apoptotic caspases in human breast carcinoma cells

Małgorzata Sztanke [1]*, Jolanta Rzymowska [2], Krzysztof Sztanke [3]

[1] Chair and Department of Medical Chemistry, Medical University, 4A Chodźki Street, 20-093 Lublin, Poland
[2] Department of Biology and Genetics, Medical University, 4A Chodźki Street, 20-093 Lublin, Poland
[3] Laboratory of Bioorganic Synthesis and Analysis, Chair and Department of Medical Chemistry, Medical University, 4A Chodźki Street, 20-093 Lublin, Poland
* Correspondence: malgorzata.sztanke@umlub.pl

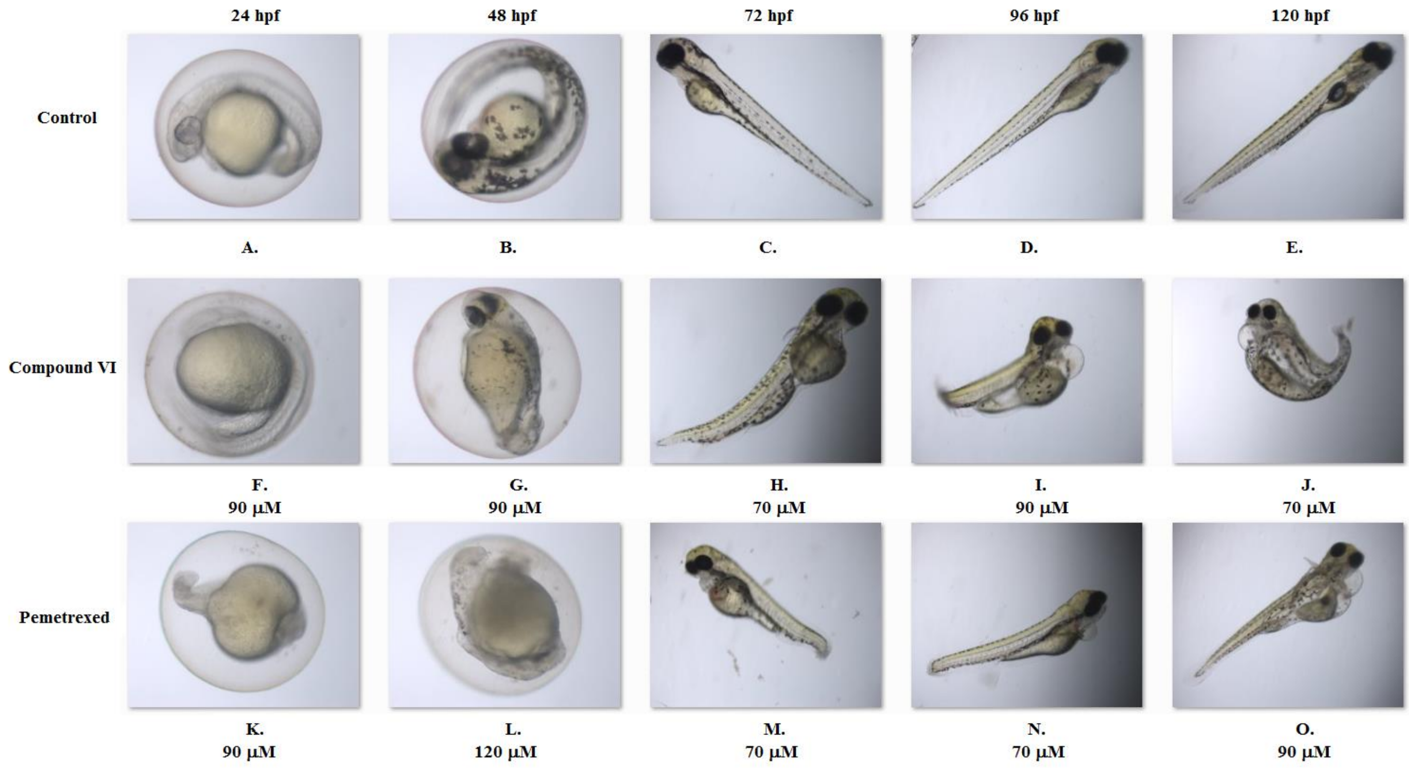

Figure S1. The representative 1-, 2-, 3-, 4- and 5-day-old zebrafish from the control group (**A-E**) as well as from groups exposed to the highest concentrations of the compound **VI** (**F-J**) or pemetrexed (**K-O**). Embryos in a control group with well-developed head and tail and clearly visible somites (**A-B**). Larvae in a control group with the normal body structure, well-developed craniofacial and tail regions as well as the normal pericardium, yolk sac and swim bladder (**C-E**). The most serious phenotypic abnormalities: embryo coagulation (**L**), undeveloped head and tail (**K**), lack of somites (**F**, **G**), pericardial oedema (**H-J**, **M-O**), yolk sac oedema (**H-J**, **M-O**), uninflated swim bladder (**J**, **O**), spine deformation (**H-J**), tail defect (**G**, **H**, **J**, **M**), that were observed in embryos and larvae treated with the compound **VI** or pemetrexed.
